# Supplementary material for: A sparse optimization approach to infinite infimal convolution regularization
Source: Numer Math (Heidelb). Author manuscript; Available in PMC 2026 Oct 16. (PMC7619587; doi:10.1007/s00211-024-01439-2)
Supplement: Supplementary file 1 [file EMS218481-supplement-Appendices.pdf]

# A Measures with values in Banach spaces

In this section we give the necessary definitions and results about measures with values in Banach spaces that are needed in this paper and we formulate them in our setting. We refer to [30, 53] to a more detailed overview about the existing theory.

## A.1 $L^p$-functions with values in Banach spaces

Let $d \in \mathbb{N}$ and $X$ be a Banach space. Given a non-empty compact set $S \subset \mathbb{R}^d$, $\mu \in \mathcal{M}_+(S)$ a Radon measure on $S$ and $p \in [1, \infty)$ we denote by $L^p((S, \mu); X)$ the space of $L^p$ functions with values in $X$ defined as

$$L^p((S, \mu); X) = \left\{ u : S \to X \text{ measurable } : \int_S \|u(s)\|_X^p \, d\mu(s) < \infty \right\} , \tag{A.1}$$

where we say that a function $u : S \to X$ is measurable if it is the $\mu$-a.e. limit of a sequence of simple functions; see [53, Section 2.3] for more details. Note also that each $u \in L^p((S, \mu); X)$ is $p$-integrable in the sense of Bochner. It is well-known that the space $L^p((S, \mu); X)$ is a Banach space with the norm

$$\|u\|_{L^p((S,\mu);X)} = \left( \int_S \|u(s)\|_X^p \, d\mu(s) \right)^{1/p} .$$

## A.2 Definition and Radon–Nikodým property

We will consider finite Radon measures defined in the compact set $S \subset \mathbb{R}^d$ with values in $X$ that is the dual of a separable Banach space $X_*$ and we denote this set as $\mathcal{M}(S;X)$. The formal definition is similar to the classical notion of measure with values in $\mathbb{R}$. Denoting by $\Sigma$ the Borel $\sigma$-algebra of $S$ a function $\mu : \Sigma \to X$ is a Borel measure if

i) $\mu(\emptyset) = 0$

ii) For any countable family $(E_i)_{i=1,}^{\infty}$ of pairwise disjoint Borel sets we have that

$$\mu\left(\bigcup_{i=1}^{\infty} E_i\right) = \sum_{i=1}^{\infty} \mu(E_i), \tag{A.2}$$

where the limit in (A.2) is taken with respect to the strong topology of $X$.

The classical definition of variation can be easily extended to measures with values in Banach spaces. Given a Borel measure $\mu$ and $E \in \Sigma$ we define

$$|\mu|(E) = \sup\left\{\sum_{i=1}^{n} \|\mu(A_i)\|_X : \ n \in \mathbb{N},\, (A_i)_{i=1}^{n} \text{ partition of } E\right\}. \tag{A.3}$$

We denote by $\mathcal{M}(S;X)$ the set of Borel measures with finite variation and we call their elements Radon measures. It can be shown that for $\mu \in \mathcal{M}(S;X)$, $|\mu|$ belongs to the set of finite positive Borel measures $\mathcal{M}_+(S)$ [30, Proposition 9] and $\mathcal{M}(S;X)$ is a Banach space with the total variation norm defined as $\|\mu\|_{\mathcal{M}(S;X)} = |\mu|(S)$ for every $\mu \in \mathcal{M}(S;X)$. We remark that every $\mu \in \mathcal{M}(S;X)$ is regular, in the sense that $|\mu|$ is regular in the classical sense, being a finite positive Borel measure defined on a compact set in $\mathbb{R}^d$. Moreover, for every $\mathcal{M}(S;X)$ we have $\mu \ll |\mu|$ in the sense that for every Borel set $E \subset S$ such that $|\mu|(E) = 0$ it holds that $\mu(E) = 0$.

**Remark A.1.** We point out that the total variation norm defined above can be replaced by the total semi-variation [30] to endow the space $\mathcal{M}(S;X)$ with an alternative Banach space structure. As we consider the integral of strongly measurable functions with respect to measures in $\mathcal{M}(S;X)$ the natural norm to be considered is the total variation norm. We refer to [30, 53] for the definition of the total semi-variation and the corresponding theory of weak integration.

In the next proposition we deal with vector measures constructed as

$$\mu(E) = \int_E u(s)\, d\sigma(s)\,, \tag{A.4}$$

for all $E \subset S$ Borel, where $\sigma \in \mathcal{M}_+(S)$ and $u \in L^1((S,\sigma);X)$. Note that the integral above is defined as the Bochner integral of $u \in L^1((S,\sigma);X)$. Following the notation for classical measures, given $\mu \in \mathcal{M}(S;X)$ defined as in (A.4) we write $\mu = u\sigma$. Moreover, we call $u$ the density of $\mu$ with respect to $\nu$ and we denote it by $u = \dfrac{\mu}{\sigma}$. Note that $u$ is unique $\sigma$ almost everywhere due to [30, Corollary 5].

**Proposition A.2.** *Let $\Sigma$ be the Borel $\sigma$-algebra of $S$, $\sigma \in \mathcal{M}_+(S)$ and $u \in L^1((S,\sigma);X)$. Then, the map $\mu : \Sigma \to X$ defined in (A.4) belongs to $\mathcal{M}(S;X)$ and its variation is given by*

$$|\mu|(E) = \int_E \|u(s)\|_X \, d\sigma \tag{A.5}$$

*for every $E \subset S$ measurable set. In particular, $\|u(s)\|_X > 0$ for $|\mu|$-a.e. $s \in S$. Moreover, $\mu \ll |\mu|$ and*

$$\mu = \hat{u}|\mu|\,, \tag{A.6}$$

*where $\hat{u} \in L^1((S,|\mu|);X)$ is defined as $\hat{u}(s) = \frac{u(s)}{\|u(s)\|_X}$ for $|\mu|$-a.e. $s \in S$.*

*Proof.* First the verification that $\mu$ belongs to $\mathcal{M}(S;X)$ is straightforward and (A.5) follows directly from Proposition 5.4 in [53]. Notice that by the properties of the Bochner integral we have the following inequality

$$\|\mu(E)\|_X \leq \int_E \|u(s)\|_X \, d\sigma(s) = |\mu|(E) \tag{A.7}$$

implying that $\mu \ll |\mu|$. We now show (A.6). Indeed, thanks to (A.5) we first notice that $\|u(s)\|_X > 0$ for $|\mu| - a.e.\ s \in S$. Thus, for every measurable set $E$ there holds

$$\int_E \frac{u(s)}{\|u(s)\|_X} \, d|\mu|(s) = \int_E u(s) \, d\sigma(s) = \mu(E) \,,$$

as we wanted to prove. □

An important property for measures with values in a Banach space $X$ is the validity of the Radon–Nikodým theorem.

**Definition A.3** (Radon–Nikodým property)**.** The Banach space $X$ has the Radon–Nikodým property if for every set $\Omega$, every $\sigma$-algebra $\Sigma$ on $\Omega$ and every measure $\mu : \Sigma \to \mathbb{R}$ of bounded variation such that $\mu \ll \sigma$ for $\sigma : \Sigma \to \mathbb{R}$ a finite positive measure, there exists $u \in L^1((\Omega, \sigma); X)$ such that $\mu = u\sigma$.

The Radon–Nikodým property is true for separable dual Banach spaces due to the classical Dunford–Pettis theorem, see Theorem 1 [30, p. III.3].

**Theorem A.4** (Dunford–Pettis)**.** *Separable dual Banach spaces have the Radon–Nikodým property.*

We conclude this section with the Lebesgue decomposition theorem adapted to measures with values in Banach spaces [30, Theorem 9].

**Theorem A.5** (Lebesgue decomposition theorem)**.** *Given $\mu \in \mathcal{M}(S;X)$ and $\sigma \in \mathcal{M}_+(S)$, there exist unique $\mu^a, \mu^s \in \mathcal{M}(S;X)$ such that*

- $\mu^a \ll \sigma$,
- $\varphi \circ \mu^s \in \mathcal{M}(S)$ *and* $\sigma$ *are mutually singular for every* $\varphi \in X^*$,
- $\mu = \mu^a + \mu^s$.

*Additionally, $|\mu| = |\mu^a| + |\mu^s|$, and $|\mu^s|$ and $\sigma$ are mutually singular.*

## A.3 Duality theory

Define the space $C(S;X)$ to be the set of continuous functions on $S$ with values in $X$ endowed with the norm topology given by

$$\|T\|_{C(S;X)} := \sup_{s \in S} \|T(s)\|_X \tag{A.8}$$

for $T \in C(S;X)$. Here we are interested in the identification of $C(S;X)^*$ with the space of vector measures $\mathcal{M}(S;X^*)$, if $S$ is a non-empty compact set of $\mathbb{R}^d$. This is a classical result due to Singer [56] that we state here for the reader's convenience. See also, for instance, [44] or [30, Section 6.5]. Note that since $S$ is non-empty and compact, the space $\mathcal{M}(S;X^*)$ coincides with the space of regular $X^*$-valued Borel measures of finite total variation.

**Theorem A.6.** *Let $S$ be a compact Hausdorff space and $X$ a Banach space. Then, there exists an isometric isomorphism $T$ between $\mathcal{M}(S;X^*)$ and $C(S;X)^*$ such that*

$$T(\mu)(f) = \int_S f \, d\mu, \quad \text{for every } \ \mu \in \mathcal{M}(S,X^*),\ f \in C(S;X)$$

*and*

$$\|\mu\|_{\mathcal{M}(S,X^*)} = \|T(\mu)\|_{C(S,X)^*} = \sup_{\|f\|_{C(S;X)} \leq 1} \int_S f \, d\mu \,, \quad \text{for every } \ \mu \in \mathcal{M}(S,X^*) \,.$$

In light of Theorem A.6 we say that a sequence of regular $X^*$-valued vector measures on $S$ denoted by $\{\mu^n\}_n$ weakly* converges to $\mu$ if

$$\lim_{n\to+\infty} \int_S f\, d\mu^n = \int_S f\, d\mu \quad \text{for every } f \in C(S; X)\,. \tag{A.9}$$

We also have the following remark.

**Remark A.7.** Theorem A.6 and the definition of weak* convergence in (A.9) imply that the total variation norm of a regular $X^*$-valued Borel measure on $S$ is weakly-* lower semicontinuous. Moreover choosing $f(s) = v \in X$ in (A.9) we obtain that

$$\lim_{n\to+\infty} \langle \mu^n(S), v\rangle = \lim_{n\to+\infty} \int_S v\, d\mu^n = \int_S v\, d\mu = \langle \mu(S), v\rangle$$

for every $v \in X$, implying that $\mu^n(S)$ converges weakly* to $\mu(S)$.

# B Fractional-Laplacian-type operators

In this section of the appendix we recall basic results and definitions about fractional Sobolev spaces, fractional Laplacian operators as well as necessary functional analytic tools required in the paper. We also consider the energies obtained as the $L^2$-norm of fractional-Laplacian-type and characterize the extremal points of their balls. For more details about fractional Sobolev spaces and fractional Laplacians we refer to [29].

## B.1 Periodic $L^2$-functions

For $q \in \mathbb{N}$ we denote by $T^q$ the $(2\pi)$-periodic torus defined by a quotient procedure as $(\mathbb{R}/2\pi\mathbb{Z})^q$. We denote by $L^2(T^q)$ the set of $L^2$ functions on $T^q$ defined as

$$L^2(T^q) = \left\{ v : T^q \to \mathbb{R} \text{ measurable} : \int_{T^q} |v(x)|^2\, dx < \infty \right\}. \tag{B.1}$$

We identify the space $L^2(T^q)$ with the space of equivalence classes of $[0, 2\pi)^q$-periodic functions $v : \mathbb{R}^q \to \mathbb{R}$ such that

$$\int_{(0,2\pi)^q} |v(x)|^2\, dx < \infty\,. \tag{B.2}$$

We endow $L^2(T^q)$ with a Hilbert structure by means of the scalar product

$$\langle v, w\rangle_{L^2(T^q)} = \int_{(0,2\pi)^q} v(x) w(x)\, dx \quad \text{for } v, w \in L^2(T^q) \tag{B.3}$$

inducing the norm

$$\|v\|_{L^2(T^q)} = \sqrt{\int_{(0,2\pi)^q} |v(x)|^2\, dx}\,. \tag{B.4}$$

The space $L^2(T^q)$ is a separable Hilbert space and its predual is $L^2(T^q)$ itself. We also introduce the $L^2$-periodic functions with zero mean, defined as

$$L^2_\circ(T^q) = \left\{ u \in L^2(T^q) : \int_{(0,2\pi)^q} v(x)\, dx = 0 \right\}. \tag{B.5}$$

Notice that $L^2_\circ(T^q)$ is a closed subspace of $L^2(T^q)$. Therefore, $L^2_\circ(T^q)$ is a separable Hilbert space as well.

## B.2 Fourier series of periodic $L^2$-functions

For $m \in \mathbb{Z}^q$ we define the $m$-th Fourier coefficient of $v \in L^2(T^q)$ as

$$\mathcal{F}v(m) = \hat{v}(m) = \frac{1}{(2\pi)^q} \int_{(0,2\pi)^q} v(x) e^{-ix\cdot m} \, dx \,. \tag{B.6}$$

It is standard to show that for every $v \in L^2(T^q)$ the Fourier series $\sum_{m\in\mathbb{Z}^q} \hat{v}(m) e^{ix\cdot m}$ converges to $v$ in $L^2(T^q)$ and we write $v(x) = \sum_{m\in\mathbb{Z}^q} \hat{v}(m) e^{ix\cdot m}$. Moreover, Parseval's theorem holds for every $v, w \in L^2(T^q)$, i.e.,

$$\langle v, w \rangle_{L^2(T^q)} = (2\pi)^q \langle \hat{v}, \hat{w} \rangle_{\ell^2(\mathbb{Z}^q)} \,. \tag{B.7}$$

## B.3 Periodic fractional Sobolev functions

We now define fractional Sobolev functions on the periodic domain $T^q$ for $q \in \mathbb{N}$.

**Definition B.1** (Fractional Sobolev functions on periodic domains)**.** Given a function $v : T^q \to \mathbb{R}$ and $s \geq 0$, we say that $v \in H^s(T^q)$ if $v \in L^2(T^q)$ and

$$[v]^2_{H^s(T^q)} := \sum_{m\in\mathbb{Z}^q} |m|^{2s} |\hat{v}(m)|^2 < \infty \,. \tag{B.8}$$

The space $H^s(T^q)$ is a Hilbert space with the scalar product defined as

$$\langle v, w \rangle_{H^s(T^q)} = \langle v, w \rangle_{L^2(T^q)} + \sum_{m\in\mathbb{Z}^q} |m|^{2s} \hat{v}(m) \overline{\hat{w}(m)} \tag{B.9}$$

inducing a norm defined as $\|v\|_{H^s(T^q)} = \sqrt{\|v\|^2_{L^2(T^q)} + [v]^2_{H^s(T^q)}}$.

**Remark B.2.** Notice that for $s = 0$ the space $H^s(T^q)$ is isomorphic to $L^2(T^q)$ and $\|v\|_{H^0(T^q)} = \sqrt{1 + (2\pi)^{-q}} \|v\|_{L^2(T^q)}$ for every $v \in L^2(T^q)$.

## B.4 The fractional Laplacian

We define the fractional Laplacian in periodic domains as follows.

**Definition B.3** (Fractional Laplacian)**.** Given $v \in H^{2s}(T^q)$ and $s \in (0, 1]$ we define the fractional Laplacian of $v$ as

$$(-\Delta^s) v(x) = \sum_{m\in\mathbb{Z}^q} |m|^{2s} \hat{v}(m) e^{ix\cdot m} \,. \tag{B.10}$$

**Remark B.4.** According to the previous definitions it is easy to check that $(-\Delta^s)v \in L^2(T^q)$ with

$$\|(-\Delta^s)v\|_{L^2(T^q)} = (2\pi)^{q/2} [v]_{H^{2s}(T^q)} \,. \tag{B.11}$$

Using Parseval's theorem it is easy to show that the following version of the Poincaré inequality holds.

**Theorem B.5** (Poincaré inequality)**.** *For every* $0 \leq s \leq 1$ *there holds*

$$\|v\|_{L^2(T^q)} \leq \|(-\Delta^s)v\|_{L^2(T^q)} \tag{B.12}$$

*for every* $v \in H^{2s}(T^q)$ *with zero mean.*

### B.5 Extremal points of $L^2$-balls of fractional-Laplacian-type operators

In this paper we will consider $L^2$-norms of fractional-Laplacian-type operators. This section is devoted to characterize the extremal points of their $L^2$ balls. Let $f : \mathbb{Z}^q \to [0, \infty)$ be a function such that

i) For every $m \neq 0$ it holds that $f(m) > 0$.

We consider the convex, positively one-homogeneous functional $J_f : L^2(T^q) \to [0, \infty)$ defined as

$$J_f(v) := \sqrt{\sum_{m\in\mathbb{Z}^q} f(m)|\hat{v}(m)|^2}\,. \tag{B.13}$$

We now characterize the extremal points of the unit ball of $J_f$ in $L^2_\circ(T^q)$.

**Lemma B.6.** *Consider the set $B = \{v \in L^2_\circ(T^q) : J_f(v) \le 1\}$. We have that*

$$\mathrm{Ext}(B) = \left\{v \in L^2_\circ(T^q) : J_f(v) = 1\right\}\,. \tag{B.14}$$

*Proof.* First note that $J_f(v) > 0$ whenever $v \neq 0$ thanks to Assumption $i)$ of $f$. Setting

$$\mathscr{B} = \left\{v \in L^2_\circ(T^q) : J_f(v) = 1\right\}\,, \tag{B.15}$$

we want to prove that $\mathrm{Ext}(B) = \mathscr{B}$. We first proceed to prove the inclusion $\mathscr{B} \subset \mathrm{Ext}(B)$. Given $v \in \mathscr{B}$ consider the convex combination

$$v = \lambda v_1 + (1-\lambda)v_2 \tag{B.16}$$

with $\lambda \in (0, 1)$ and $v_1, v_2 \in B$. Applying the functional $J_f$ to both sides of (B.16), using the convexity of $J_f$ and that $v_1, v_2 \in B$ we obtain that $\lambda J_f(v_1) + (1-\lambda)J_f(v_2) = 1$ implying that $J_f(v_1) = J_f(v_2) = 1$. Therefore, from (B.16) we get

$$1 = \lambda^2 \sum_{m\in\mathbb{Z}^q} f(m)|\hat{v}_1(m)|^2 + (1-\lambda)^2 \sum_{m\in\mathbb{Z}^q} f(m)|\hat{v}_2(m)|^2 + 2\lambda(1-\lambda)\mathrm{Re}\sum_{m\in\mathbb{Z}^q} f(m)\overline{\hat{v}_1(m)}\hat{v}_2(m)$$

implying, since $\lambda \neq 0$ and $\lambda \neq 1$, that

$$\mathrm{Re}\sum_{m\in\mathbb{Z}^q} f(m)\overline{\hat{v}_1(m)}\hat{v}_2(m) = 1\,.$$

Recalling that $J_f(v_1) = J_f(v_2) = 1$, a simple contradiction argument that uses the sharpness of Cauchy-Schwarz inequality shows that $\hat{v}_1(m) = \hat{v}_2(m)$ for every $m \in \mathbb{Z}^q$. In particular, $v_1 = v_2 = v$ implying that $v$ is an extremal point for $B$.

Conversely, let us show that $\mathrm{Ext}(B) \subset \mathscr{B}$. Suppose that such inclusion does not hold true. Then, there exists $v \in \mathrm{Ext}(B)$ such that $J_f(v) \neq 1$. In particular, since $v \in B$, there exists $\varepsilon > 0$ such that $J_f(v) < 1-\varepsilon$. Consider $w \in L^2_\circ(T^q) \setminus \{0\}$ such that $J_f(w) \le \frac{\varepsilon}{2}$. Notice that such $w$ exists since $\mathrm{dom}\, J_f \neq \{0\}$ and $J_f$ is positively one-homogeneous. Consider then the convex combination

$$v = \frac{1}{2}(v+w) + \frac{1}{2}(v-w)\,. \tag{B.17}$$

Since $w \neq 0$, we immediately have that $v + w \neq v - w$. Moreover

$$J_f(v \pm w) \le J_f(v) + J_f(w) < 1\,, \tag{B.18}$$

showing that $v$ is not an extremal point. □

If $f$ is strictly positive, then we can characterize the extremal points of $J_f$ in $L^2(T^q)$ as follows.

**Lemma B.7.** *Suppose that $f$ satisfies*

*i') For every $m \in \mathbb{Z}^q$ it holds that $f(m) > 0$.*

*Then given the set $\tilde{B} = \{v \in L^2(T^q) : J_f(v) \le 1\}$ we have that*

$$\mathrm{Ext}(\tilde{B}) = \left\{v \in L^2(T^q) : J_f(v) = 1\right\}\,. \tag{B.19}$$

Since the proof follows closely the one of Lemma B.6 we omit it.
